# Antimicrobial Stewardship Program in a Low-Middle Income Country: Impact of an Antibiotic Guideline for Neonatal Early-Onset Sepsis

**DOI:** 10.3390/antibiotics15070639

**Published:** 2026-06-26

**Authors:** Minh T. N. Le, Anh T. Do, Ha T. Pham, Cuc T. Nguyen, Tung V. Cao, Ha T. H. Nguyen, Hoa D. Vu, Hang T. Nguyen, Anh V. Nguyen, Hung C. Dao, Tung A. Tran, Jennifer Le

**Affiliations:** 1 Vietnam National Children’s Hospital, Hanoi 11500, Vietnam; nguyetminhlt@nch.gov.vn (M.T.N.L.); thuyanh@nch.gov.vn (A.T.D.); hapt@nch.gov.vn (H.T.P.); tungcv@nch.gov.vn (T.V.C.); honghaduocviennhi@gmail.com (H.T.H.N.); vudinhhoa@gmail.com (H.D.V.); hang.nt@nch.gov.vn (H.T.N.); vietanh24@nch.gov.vn (A.V.N.); hungdc@nch.gov.vn (H.C.D.); anhtungtran12@nch.gov.vn (T.A.T.); 2 National Drug Information and Adverse Drug Reaction Monitoring Centre, Hanoi University of Pharmacy, Hanoi 11021, Vietnam; cucnt@hup.edu.vn; 3 Skaggs School of Pharmacy and Pharmaceutical Sciences, University of California at San Diego, La Jolla, CA 92093, USA; 4 Tylan Health, El Monte, CA 91732, USA

**Keywords:** antibiotic, antimicrobial, stewardship program, neonate, infant, early-onset sepsis, low- and middle-income countries

## Abstract

**Background/Objectives**: Initiation of empiric antibiotic therapy for neonatal early-onset sepsis (EOS) is prudent to prevent morbidity and mortality, particularly in low- and middle-income countries (LMICs). Inappropriate or prolonged antibiotic exposure in neonates is associated with poor clinical outcomes. Antimicrobial stewardship programs (ASPs) have been shown to optimize antibiotic use, but data from LMICs are limited. In this study, we aimed to evaluate adherence to a locally developed and adopted guideline for antibiotic use in EOS. **Methods**: We conducted a retrospective before-and-after study during the pre- (June 2024–January 2025) and post-implementation (May–December 2025) of ASP guideline for EOS. The intervention involved consolidating best practices—previously shared verbally and applied variably into a locally united written guideline, then provide training to neonatologists, pharmacists, and nurses. Adherence to best practices was evaluated by indication, dosing, timing, and duration of antibiotic therapy. **Results**: In a cohort of 388 neonates with EOS (i.e., 205 pre- and 183 post-implementation), the median gestational age was 38 (IQR: [37–39]) weeks, with the median birthweight of 3000 (IQR: [2800–3400]) grams, and 63% were male. The total adherence improved from 2.0% to 65.6% (*p* < 0.001) from pre- to post-implementation of ASP. In the post period, adherence rates were 96.7% for empiric antibiotics indication, 95.6% for antibiotics indication after culture results are obtained, 88.5% for antibiotic dosing, 83.1% for timely antibiotic initiation, and 89.1% for appropriate discontinuation of antibiotics. The median days of therapy and length of therapy significantly decreased by 139 per 1000 patient-days, from 1806 (IQR: [1556–2083] to 1667 (IQR: [1400–2000]; *p* < 0.001)] patient-days; and from 1000 (IQR: [875–1000]) to 875 (IQR: [769–1000]; *p* < 0.001) patient-days in the pre- versus the post-implementation, respectively. Median length of hospitalization of 8 [7–12] days and recovery (~93%) from EOS were similar pre- and post-implementation. **Conclusions**: The results support the effectiveness of ASP implementation in improving guideline adherence and reducing antibiotic exposure among neonates with EOS in low-resource settings. In-hospital clinical outcomes, including mortality at discharge, were similar between periods; however, further studies with longer follow-up are needed to better evaluate clinical outcomes.

## 1. Introduction

Infections remain one of the leading causes of neonatal morbidity and mortality, particularly in low- and middle-income countries (LMICs), where sepsis-attributable neonatal mortality rates vary from 10 to 29% [1,2]. In addition, the growing burden of antimicrobial resistance (AMR) poses an increasing challenge to antibiotic effectiveness and progress in reducing mortality, especially in LMICs, where an estimated 140,000–214,000 neonatal deaths per year are related to AMR [3].

Data from LMICs show an overlap between the bacterial etiologies of early-onset sepsis (EOS) and late-onset sepsis (LOS), in contrast to the clear etiological differences in high-income countries [4]. A systematic review and meta-analysis of neonatal sepsis pathogens in developing countries found *Klebsiella* spp., *Staphylococcus aureus*, coagulase-negative staphylococci (CoNS), *Escherichia coli*, as leading causes of sepsis [5]. In LMICs, the most frequently reported causes of neonatal infection are Gram-negative bacteria such as *Klebsiella pneumoniae*, *Escherichia coli*, *Acinetobacter baumannii*, and Gram-positive bacteria *Staphylococcus aureus* [4]. In Vietnam National Children’s Hospital (VNCH), a retrospective cross-sectional study reported that Gram-negative bacteria accounted for 75.7% of all infection cases, including 50% of EOS. Moreover, this study highlighted significant resistance of Gram-negative bacteria at VNCH to third-generation cephalosporins, carbapenem and considerable resistance to clindamycin and oxacillin [6].

Since early clinical signs of sepsis in neonates are nonspecific, initiation of empirical antibiotic therapy is common practice [7]. Antibiotics are among the most frequently prescribed drugs in neonatal intensive care units (NICUs), but this practice carries substantial risks. Studies have shown that inappropriate or prolonged antibiotic exposure in neonates is associated not only with increased AMR but also with adverse short- and long-term outcomes, including altered hepatic and renal function, increasing risk of comorbidities and disrupting the microbiota [8]. These findings emphasize the importance of balancing the benefits of early treatment against the harms of unnecessary antibiotic exposure.

Antimicrobial stewardship programs (ASPs) are increasingly advocated as a strategy to optimize antibiotic use while minimizing resistance. A meta-analysis of ASP in neonates documented that implementations (including guidelines, policies and calculators) reduce antibiotic use and duration of therapy, without increasing sepsis-related mortality [9]. This research also showed a significant reduction in the proportion of neonates receiving initial antibiotic treatment in the NICU, duration, and the proportion of patients receiving antibiotics for more than 5 days. A 2025 study in India also demonstrated significant decrease in antibiotic duration and the use of empirical antibiotic [10]. Despite these encouraging findings, most evidence is collected from high-income countries, and there are limited data from LMIC settings where antibiotic overuse and AMR burden are often greater.

At VNCH, prior to the ASP in neonatal EOS, best practices were shared verbally and applied variably among clinicians. Therefore, when ASP on neonatal EOS was commenced, an institutional guideline was developed and training was provided to neonatologists, pharmacists, and nurses. In this study, our primary objective was to evaluate adherence to the institutionally-developed EOS guideline under ASP strategies in neonatal care within a LMIC setting. In addition, our secondary objective was to evaluate treatment outcomes, including total length of hospital and NICU stay, recovery from infection, and mortality.

## 2. Results

**Figure 1:**
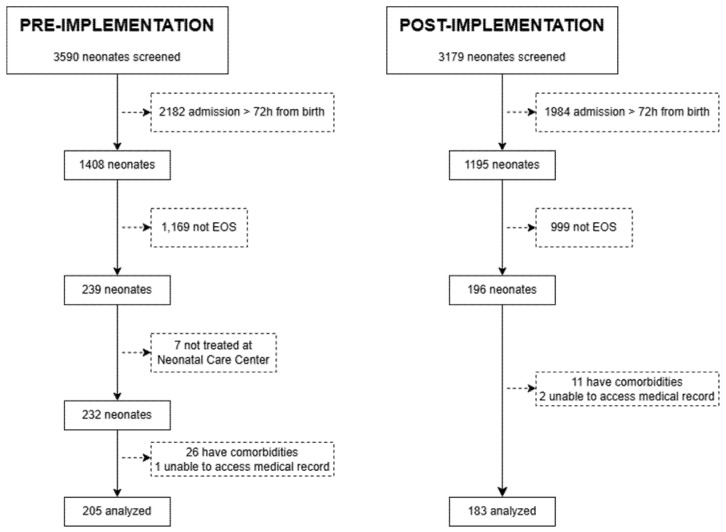
Consort Diagram for Retrospective Before-and-After Study.

A total of 6769 neonates were screened for eligibility. The final sample included 388 neonates, of whom 205 were included in the pre-implementation and 183 in the post-implementation groups (Figure 1).

One-fifth of the study patients (77/388, 19.8%) were premature neonates, 5.2% (20/388) of them having very low to extremely low birth weight and a half (219/388, 56.4%) admitted to NICU. Baseline demographic data were comparable between the pre- and post-implementation groups, including gestational age, proportion in premature groups, birth weight, gender and NICU admission rate (Table 1). The rate of admission from referring facilities was high in both groups, 80.5% compared to 71.0%, (*p* = 0.04) in pre- and post-implementation groups, respectively.

The appropriateness of antibiotic use included total adherence, indication adherence, dose adherence, timing and duration adherence (Table 2). Overall adherence to best practices improved from 2% inter pre-implementation period to 65.6% in the post-implementation period (*p* < 0.001). Improvements were observed across all four predefined criteria, including empiric indication (82.9% vs. 96.7%, *p* < 0.001), post-culture indication (87.8% vs. 95.6%, *p* = 0.01), dosage (12.2% vs. 88.5%, *p* < 0.001), timing of first dose (29.3% vs. 83.1%, *p* < 0.001), and discontinuation of antibiotics (67.8% vs. 89.1%, *p* < 0.001). The best improvement was noted in appropriate antibiotic dose, increasing from 12.2% to 88.5% (*p* < 0.001). The most common empiric antibiotics were ampicillin and tobramycin, used in more than 67% of post-implementation patients.

Total days of therapy (DOT) for each antibiotic, and the length of total antibiotic therapy (LOT) at the end of therapy were also analyzed. DOT and LOT decreased significantly in the post-implementation period. The median DOT decreased from 1806 [1556–2083] to 1667 [1400–2000] per 1000 patient-days (*p* < 0.001), while the median LOT decreased from 1000 [875–1000, range: 67–1000] to 875 [769–1000] per 1000 patient-days (*p* < 0.001). In addition, the proportion of antibiotic courses exceeding 5 days declined significantly from 85.4% to 73.2% (*p* = 0.005). Time from first blood sample result to discharge didn’t change significantly in both positive and negative culture group.

No significant differences in treatment outcomes were observed between the pre- and post-implementation groups (Table 3). Most patients (>90%) recovered from EOS and hospital days were similar pre- and post-implementation. All positive-culture patients were microbiologically cured at discharge.

Mortality at discharge, including cases discharged at the family’s request, was comparable between groups, with 11 patients (5.4%) in pre-implementation group and 8 patients (4.4%) in post-implementation group (*p* = 0.83). Given the small number of mortality events (*n* = 19), the study had limited statistical power to detect potentially meaningful differences in mortality between the periods.

## 3. Discussion

Vietnam continues to have a high burden of AMR among Asian countries; to combat this, ASPs have been broadly adopted and shown to be effective [11]. Newborns are particularly vulnerable to AMR, which contributes substantially to premature mortality; thus, they should be prioritized in ASP efforts [12]. To our knowledge, this study was the first in Vietnam to evaluate adherence to the guideline specifically for neonatal EOS within the framework of ASP implementation.

Our study demonstrated that the adoption of the neonatal EOS guideline under the framework of ASP resulted in significant improvement in the total adherence from pre- to post-implementation period, as observed in antibiotic prescription using all four criteria, incorporating antibiotic selection, dosing, timing, and duration [9,13,14]. Total adherence in the pre-ASP period was very low (2%), possibly due to unfamiliarity with the new standardized dosing regimen. The most significant improvement after ASP interventions was observed in dosing, which could be attributed to frequent interventions by the ASP team and acceptance from the neonatal unit clinicians [15]. A full commitment from the ASP team, with the involvement of clinical pharmacists and health care professionals, to enhance antibiotic prescribing was pivotal to the successful implementation of our EOS guideline, as reported in previous studies [15,16,17]. Data on the total DOT and LOT per 1000 patient-days also decreased significantly from the pre- to post-periods, consistent with previous studies and highlighting the impact of ASP interventions in the neonatal population [18,19,20].

Almost all patients had blood cultures obtained; notably, the rate of culture-negative results was high across both periods, with 94.2% and 91.4% in the pre- and post- implementation periods, respectively. This continues to pose a significant challenge in managing neonatal EOS, and underscores the necessity of applying maternal history, clinical presentations and laboratory biomarker evidence of infection to guide antibiotic therapy [21,22]. Furthermore, our data indicated no difference in the duration of antibiotic treatment, measured from the time of the first blood sample result until the patient’s discharge, between the pre- and post-ASP periods, in both groups of negative and positive cultures. This highlights a demand for improvement in ASP, specifically in better management of unnecessarily prolonged antibiotic exposure in EOS patients [23,24]. However, in our study, the number of patients requiring more than 5 days of antibiotic therapy decreased significantly in the post-implementation period (*p* = 0.02), indicating a positive change in the prescribing practices of neonatologists. No statistically significant differences were observed in the secondary outcomes, including length of hospital stay, NICU stay, recovery at discharge, and mortality. Mortality was numerically lower in the post-implementation period (4.4% vs. 5.4%); however, only 19 deaths occurred in the entire cohort, limiting the statistical power to detect significant differences between study periods. Although maternal risk factors for EOS were similar across the two periods, we still highlight the need to document neonatal patient characteristics in relation to treatment outcomes in future studies to identify risk factors that may affect recovery time, as reported in previous studies [25,26].

Early discontinuation of empiric antibiotics is a cornerstone of antimicrobial stewardship in neonatal EOS, where many infants are treated empirically despite ultimately having culture-negative evaluations, as evident in our study, with more than 90% culture negativity. A standard course of antibiotic therapy for culture-negative EOS was 5 to 7 days in our study especially if clinical signs and laboratory findings remained indicative of possible infection; however, a standard duration of therapy should be used cautiously, and individualized therapy is recommended to address this issue [18,20,27]. Patient-specific duration approach is particularly critical in LMICs like Vietnam, where neonatal sepsis burden is high and AMR is accelerating. Bacterial pathogens for EOS in LMICs differ from those in high-income countries, with organisms with a high potential for antibiotic resistance such as *Escherichia coli*, *Klebsiella pneumoniae*, and *Staphylococcus aureus* being predominant [28,29]. This results in the use of broad-spectrum antibiotics that further leads to an increase in the rate of resistance, making ASP more difficult yet critical in clinical practice [6,30,31]. Additionally, more than 70% of our patients were admitted from referring facilities, which suggests an earlier exposure to antibiotics at lower-tier hospitals for initial management, along with a high rate of preterm births, requiring effective control policies for the use of antibiotics in this vulnerable population [6].

Stewardship frameworks emphasize optimizing both duration and exposure—ensuring adequate pharmacokinetic-pharmacodynamic (PK-PD) target attainment while minimizing unnecessary continuation of therapy. Foundational work highlights that ASP must couple early clinical reassessment with PK-informed dosing to safely limit antibiotic duration and reduce resistance pressure [32]. A recent study reported that adequate post-discontinuation antibiotic exposure was frequent among hospitalized neonates; this represents an underrecognized driver of total antibiotic burden and underscores the need for ASP strategies that extend beyond initial prescribing decisions [33,34]. In settings with limited diagnostic capacity and high empiric antibiotic use, implementing standardized early discontinuation pathways, supported by PK evidence of adequate drug exposure, can safely reduce unnecessary antibiotic days while preserving the effectiveness of first-line agents such as ampicillin. By aligning early discontinuation practices with exposure-driven dosing and PK-informed reassurance, ASP programs can help conserve antibiotic efficacy for EOS in LMICs, where therapeutic options are constrained and resistance threatens neonatal survival [32,35,36].

Several study limitations should be acknowledged. First, the data on prior medication use at referring facilities before admission to VNCH were not fully captured, which may have influenced decisions regarding antibiotic initiation at our institution and subsequent clinical outcomes. Second, although baseline characteristics were generally comparable between groups, the proportion of patients admitted from referring facilities differed significantly between periods. This difference may reflect variation in baseline clinical context, prior management, or previous antibiotic exposure. Additionally, we did not evaluate some important severity-related variables, including necrotizing enterocolitis and mechanical ventilation, that may have impacted clinical outcomes, especially in patients with prolonged antibiotic therapy. Finally, assessment of clinical outcomes was limited to hospitalization and discharge. Post-discharge mortality, readmissions, recurrent infections, delayed treatment failure, and clinical deterioration after antibiotic discontinuation were not evaluated. Therefore, the study was not designed to fully assess the long-term safety of reduced antibiotic exposure. Moreover, the small number of mortality events limited our ability to detect potentially meaningful differences in mortality between study periods. However, our primary objective was to ascertain antibiotic use, rather than clinical outcomes.

Our study findings demonstrate that improved adherence to four criteria for antibiotic use in EOS reflects the beneficial impact of implementing ASP. These results encourage further adoption of additional ASP activities to optimize antibiotic use in the neonatal population, especially in low-resource settings. Future research should address documentation deficiencies and propose a combination of ASP implementations for this population to achieve better outcomes.

## 4. Materials and Methods

### 4.1. Study Design and Population

We conducted a retrospective before-and-after study, pre- and post-implementation of the EOS guideline, at VNCH in Hanoi, Vietnam. The study was executed with the participation of the Antimicrobial Stewardship Committee and the Neonatal Care Center. Prior to implementation, EOS management was guided by verbally shared best practices based on various external reference sources. The intervention involved consolidating these practices into a locally adapted written guideline and providing training to physicians, pharmacists, and nurses at the Neonatal Care Center. After the development of Guideline for Antibiotic Use in Early-Onset Neonatal Infections, we assessed best-practices adherence according to predefined evaluation criteria. Following implementation, the AMS team periodically observed antibiotic use from patients’ medical records. Identified non-compliance cases were addressed and discussed in subsequent meetings.

The study included neonates admitted to VNCH within 72 h of age and diagnosed with EOS. We excluded patients with comorbidities, or congenital anomalies, or patients transferred to another department for treatment.

### 4.2. Assessments and Data Collection

Demographic and clinical data were collected for all subjects using paper medical records, including microbiological culture results, antibiotic indication, dosage, timing and duration of antibiotic therapy. Guideline adherence was evaluated according to four criteria: (1) indication was considered appropriate if it was consistent with the guideline recommendations, for both initial empirical treatment and post-culture treatment; (2) dosage adherence was defined as a deviation within ±10% of the dose recommended by the guideline; (3) timing of the first antibiotic dose within 60 min from the time of diagnosis was classified as adherent; and (4) duration adherence was defined as proper discontinuation of antibiotics, in addition total days of therapy (DOT) for each antibiotic, and the length of total antibiotic therapy (LOT) at the end of therapy. The guideline-recommended durations were determined by culture results, infection severity, and clinical response.

Eligible neonates included all infants with EOS during study period. The pre-implementation period from 1 June 2024, to 31 January 2025, was compared to the post-implementation period from 1 May 2025, to 31 December 2025; a washout period from February to April 2025 was included for guideline adoption.

### 4.3. Outcomes

The primary outcome was overall adherence to our local guideline for antibiotic use for EOS, assessed across four predefined criteria: indication, dosage, timing, and duration of antibiotic therapy. Specific for duration, DOT (representing the total sum of the days for each antibiotic) and LOT (representing the total number of days a patient receives any antibiotic per 1000 patient-days) were used because they were common metrics for pediatric hospitals’ ASP [9]. Adherence to appropriate duration of antibiotic therapy was defined as the percentage of patients who had appropriate discontinuation of antibiotic therapy per the guideline recommendation.

The secondary outcomes were treatment outcomes, including length of hospital stay, duration of neonatal intensive care unit (NICU) stay, microbiological cure, discharge status, and mortality. Microbiological cure was determined if patients had positive-culture results and at least one negative result after that. Discharge status was classified as recovered from infection, transferred to primary healthcare settings after stabilization, or death during hospitalization, which based on the neonatologists’ assessment before hospital discharge.

### 4.4. Statistical Analysis

Statistical analyses were performed using R software version 4.6.0. Continuous variables (e.g., age and weight) were expressed as means and standard deviations (for normal distribution variables), or median and interquartile range (for non-normal distribution variables), and categorical variables (e.g., gender and adherence rate) as number and percentages.

Comparisons between the pre- and post-implementation periods were conducted using the two-sided Student’s *t*-test (or the Mann-Whitney U test for non-normal distribution) for continuous variables. Categorical variables were compared using the Chi-square (χ^2^) test or Fisher’s exact test. Statistical significance was defined as a *p*-value < 0.05.

## Figures and Tables

**Table 1:** Baseline demographic and clinical variables.

	All *N* = 388	Pre-Implementation *n* = 205	Post-Implementation *n* = 183	*p*-Value
Gestational age (GA) median [IQR], (weeks) ^1^	38.0 [37.0–39.0]	38.0 [37.0–39.0]	38.1 [37.0–39.4]	0.40
Preterm (GA < 37 weeks), *n* (%)	77 (19.8)	39 (19.0)	38 (20.8)	0.76
Extremely and very preterm (GA < 32 weeks), *n* (%)	24 (6.2)	9 (4.4)	15 (8.2)	0.18
Very low birth weight (from 1000 g to <1500 g), *n* (%)	10 (2.6)	3 (1.5)	7 (3.8)	0.20
Extremely low birth weight (<1000 g), *n* (%)	10 (2.6)	6 (2.9)	4 (2.2)	0.76
Birth weight, median [IQR], (grams) ^1^	3000 [2800–3400]	3000 [2800–3400]	3100 [2800–3400]	0.29
Male gender, *n* (%)	245 (63.1)	131 (63.9)	114 (62.3)	0.82
Admission from referring facilities, *n* (%)	295 (76.0)	165 (80.5)	130 (71.0)	0.04
NICU admission, *n* (%)	219 (56.4)	122 (59.5)	97 (53.0)	0.23
Maternal history, *n* (%)				0.30
Intra-amniotic infection	9 (2.3)	2 (1.0)	7 (3.8)	
Vaginitis	76 (19.6)	43 (21.0)	33 (18.0)	
Fever during labor	13 (3.4)	8 (3.9)	5 (2.7)	
Positive Group B Streptococcus	18 (4.6)	7 (3.4)	11 (6.0)	
Amniotic fluid leakage	4 (1.0)	2 (1.0)	2 (1.1)	
Intrapartum antibiotic use	6 (1.5)	4 (2.0)	2 (1.1)	
Antibiotic history, *n* (%)				0.06
No data available	177 (45.6)	82 (40.0)	95 (51.9)	
No prior use	50 (12.9)	29 (14.1)	21 (11.5)	
Previously used	161 (41.5)	94 (45.9)	67 (36.6)	
Ampicillin/Ampicillin-Sulbactam/Amoxicillin-Clavulanic ± Aminoglycoside	99 (25.5)	56 (27.3)	43 (23.5)	
Cephalosporin 3rd, 4th generation	51 (13.1)	35 (17.1)	16 (8.7)	
Imipenem-Cilastatin/Meropenem	17 (4.4)	10 (4.9)	7 (3.8)	
Microbiological culture in neonatal patients, *n* (%) ^2,5^				0.31
Negative culture	559 (92.8)	292 (94.2)	255 (91.4)	
Positive culture	30 (7.7)	17 (8.3)	13 (7.1)	
Bacterial pathogens, *n* (%) ^5^				
Suspected contaminants ^3^	13 (3.4)	10 (4.9)	3 (1.6)	
Confirmed infection	17 (4.4)	7 (3.4)	10 (5.5)	
*Staphylococcus aureus*	4 (1.0)	2 (1.0)	2 (1.1)	
*Enterococcus faecalis*	1 (0.3)	1 (0.5)	0	
*Escherichia coli*	2 (0.5)	1 (0.5)	1 (0.5)	
*Klebsiella pneumoniae*	1 (0.3)	0	1 (0.5)	
*Acinetobacter baumannii*	1 (0.3)	0	1 (0.5)	
*Others* ^4^	8 (2.1)	3 (1.5)	5 (2.7)	

*^1^ IQR: interquartile range. ^2^ Six patients (including 5 in the pre- and 1 in the post-implementation groups) did not have cultures. ^3^ Suspected contaminants included: Staphylococcus haemolyticus, Staphylococcus cohnii, Staphylococcus epidermidis, Staphylococcus ureilyticus, Staphylococcus xylosus, Streptococcus salivarius, and Mammaliicoccus sciuri. ^4^ Others included: Candida albicans (1), Citrobacter koseri (1), Staphylococcus argenteus (1), Staphylococcus cediderisdis (1), Streptococcus beta hemolytic (1), Staphylococcus epidermidis (3). ^5^ Cultured from any anatomic site: blood (384), endotracheal aspirate (84), cerebrospinal fluid (58), and others (63).*

**Table 2:** Adherence to implementation of the neonatal early-onset sepsis guideline on appropriate use of antibiotics.

	All *N* = 388	Pre-Implementation *n* = 205	Post-Implementation *n* = 183	*p*-Value
Total adherence, *n* (%)	124 (32.0)	4 (2.0)	120 (65.6)	<0.001
Appropriate empiric treatment, *n* (%)	347 (89.4)	170 (82.9)	177 (96.7)	<0.001
First-line antibiotics (ampicillin and aminoglycoside ^1^)	240 (61.9)	116 (56.6)	124 (67.8)	
Cefotaxime	114 (29.4)	73 (35.6)	41 (22.4)	
Meropenem	31 (8.0)	15 (7.3)	16 (8.7)	
Vancomycin	11 (2.8)	5 (2.4)	6 (3.3)	
Appropriate antibiotics based on positive culture results, *n* (%)	355 (91.5)	180 (87.8)	175 (95.6)	0.01
Appropriate empiric antibiotic dose, *n* (%)	187 (48.2)	25 (12.2)	162 (88.5)	<0.001
Ampicillin	262 (80.8)	106 (65.0)	156 (96.9)	
Gentamicin	19 (79.2)	14 (77.8)	5 (83.3)	
Tobramycin	144 (46.2)	10 (6.2)	134 (89.3)	
Cefotaxime	64 (44.4)	25 (34.2)	39 (95.1)	
Meropenem	24 (77.4)	10 (66.7)	14 (87.5)	
Vancomycin	7 (63.6)	1 (20.0)	6 (100)	
Timing				
Receipt of first antibiotic dose within 1 h after diagnosis, *n* (%)	212 (54.6)	60 (29.3)	152 (83.1)	<0.001
Duration of therapy				
Discontinued antibiotic appropriately ^3^, *n* (%)	302 (77.8)	139 (67.8)	163 (89.1)	<0.001
Days of therapy per 1000 patient days, median [IQR], (days)	1714 [1429–2000]	1806 [1556–2083]	1667 [1400–2000]	<0.001
Length of therapy per 1000 patient days, median [IQR], (days)	1000 [833–1000]	1000 [875–1000] ^2^	875 [769–1000]	<0.001
Length of antibiotic therapy > 5 days, *n* (%)	309 (79.6)	175 (85.4)	134 (73.2)	0.005
Time from first blood sample result to discharge, median [IQR], (days)				
Negative culture	2.0 [1.0–6.3]	2.0 [0.0–7.0]	2.0 [1.0–6.0]	0.63
Positive culture	7.0 [2.0–10.0]	8.0 [3.0–10.0]	7.0 [2.0–10.0]	0.57

*^1^ Tobramycin was the most used aminoglycoside. ^2^ Median LOT per 1000 patient days [min–max]: 1000 [67–1000]. ^3^ Appropriate durations of antibiotic therapy according to the EOS guideline.*

**Table 3:** Treatment outcomes of neonatal early-onset sepsis.

	All *N* = 388	Pre-Implementation *n* = 205	Post-Implementation *n* = 183	*p*-Value
Length of hospital stay, median [IQR], days	8.0 [7.0–12.0]	8.0 [7.0–12.0]	8.0 [7.0–13.0]	0.90
Length of NICU stay, median [IQR], days	6.0 [4.0–8.0]	6.5 [5.0–8.0]	5.0 [4.0–8.0]	0.16
Microbiological cure, *n* (%)	30 (100%)	17 (100%)	13 (100%)	-
Discharge status, *n* (%)				0.54
Death during hospitalization	19 (4.9%)	11 (5.4%)	8 (4.4%)	0.83
Recovered	362 (93.3%)	189 (92.2%)	173 (94.5%)	0.47
Transferred	7 (1.8%)	5 (2.4%)	2 (1.1%)	0.45

## Data Availability

Data is unavailable due to ethical restrictions. Further inquiries can be directed to the corresponding author.

## Abbreviations

The following abbreviations are used in this manuscript:

EOS	early-onset sepsis
LOS	late-onset sepsis
LMICs	low- and middle-income countries
NICU	neonatal intensive care unit
IQR	interquartile range
ASP	Antimicrobial Stewardship Programs
AMR	antimicrobial resistance
VNCH	Vietnam National Children’s Hospital
DOT	days of therapy
LOT	length of therapy
